# A Sketch of the Life and Character of John Armstrong, M. D.

**Published:** 1830

**Authors:** 


ART. IX.—*A sketch of the life and character of* JOHN ARMSTRONG, M. D.

Dr. John Armstrong, one of the most zealous and illustrious cultivators of medical science—a popular teacher, an admired writer, and a successful practitioner, died on the 12th December 1829, a martyr to his professional and scientific pursuits.

Believing that some account of a man, whose writings are so extensively circulated in this country, will not be unacceptable to the public, we have selected from the Med. Chirurgical Review, the facts upon which the observations are founded, that we shall now take the liberty of presenting to our readers.

The original education of Dr. Armstrong, both medical and classical, was very limited. His natural abilities and his great industry and observation, however, soon abundantly supplied this defect, and his writings evince a very intimate acquaintance with the principles of his profession, and with subjects of more general interest. Indeed in the pursuit of a profession where knowledge can be of little use, until personal observation has directed its practical application, the want of professional learning was of comparatively little consequence to a mind like his, fitted by nature with peculiar talents for the investigation of disease.

His "Practical Illustrations of Typhus Fever," the work in which he published the principles and practice upon which his fame principally rests, was published in 1816. Two years afterward, encouraged by the favorable impression he had made upon the profession, he removed to London, and shortly after, a vacancy having occurred in the London Fever Hospital, he became a candidate for the appointment of physician to that institution. The canvass having commenced, he offered himself to the College of Physicians for examination, and a license. For some reasons, which have never been explained, he was rejected—the crisis of his fate was at hand, and the irretrievable ruin of his professional prospects must have appeared to be the inevitable result. But public feeling reacted strongly in his favor, and the event which threatened to blast his prospects was the means of accelerating his elevation. Not only was he elected physician to the institution, with a full knowledge of his rejection by the board of censors, but a great many practitioners interfered in his behalf, and materially promoted his introduction into practice. Great as Dr. Armstrong's talents certainly were, assisted also by his most interesting manners, his social virtues, and by the most consummate tact and worldly wisdom, his unexampled success in his professional pursuits, can only be explained by the strong interest excited in his behalf, in consequence of this unjust decision.

Not satisfied with having secured a very extensive and lucrative practice, he directed his attention to delivering a course of lectures, in which he proved eminently successful. His high reputation, his animated and eloquent manner, his plausible opinions and ingenious disquisitions, illustrated and defended by the results of his extensive and accurate observation, together with the practice of elucidating his lectures by numerous drawings and preparations, soon procured him a larger class of students than that of any other physician, in London.

The publication of Dr. Armstrong's Lectures is said not to have enhanced his reputation, among those members of the profession who were best qualified to judge of their merit. The cultivation of the branches of science subsidiary to medicine has so far extended the field of study, that no person however great his genius, and extensive his acquirements, can expect to maintain in a course of lectures, the reputation he may have acquired by concentrating his labours upon a single subject. Retailing, as he must to a very considerable extent, the observations, opinions and practice of others, he loses the interest of originality, and the confidence arising from personal observation.

His lectures however served to extend his reputation and practice. The proverbial facility with which the minds of youth adopt the views of their instructors, applies with peculiar force to students of Medicine. The principles of the profession, depending not upon obvious and familiar facts, but upon observations, intricate, complicated and indefinite, afford to the ingenious professor an opportunity of imposing his peculiar views to an unlimited extent. And further, the student dependent upon his preceptor's assistance in every stage of his progress, very generally becomes attached to his person and overrates his talents.

Dr. Armstrong never exhibited the marks of a sound constitution. He was always delicate, and frequently affected with a cough, which he attributed to a catarrhal affection, but which, in all probability, arose from an hereditary predisposition to consumption. From the great number and hardness of the tubercles which were found in his lungs after death, they probably had existed from childhood. That they should have been excited into such fatal activity at so late a period of his life, can be accounted for, only by the great labor which he imposed upon himself. An extensive practice, to which he paid assiduous attention—lecturing in a confined and unhealthy situation during the whole year, and frequently several times in the same day—the inhalation of putrid effluvia during the examination of morbid parts, together with the great excitement into which he worked himself in every lecture, were more than sufficient to break down a constitution in which lurked already the seeds of a fatal disease. During the last 18 months of his life, his disease in the estimation of his friends began to assume a very formidable character. In the course of the last summer, he was obliged to desist from lecturing, and retire into the country for change of air. After travelling a few weeks, he returned to London, apparently much improved in health, and in his own estimation quite well. In September he went to the north of England, his native country, and returned to the city in October much worse.

That incredulity which is characteristic of pulmonary affections, retained possession of the mind of this intelligent physician, to the last stage of his disease. He would never admit that there was any serious disease of his lungs, and even so late as Novr. authorized his friends to contradict such a report on his own special authority. He continued to ride out in his carriage until within 10 or 14 days of his death, which took place on Saturday the 12th December, in the 46th year of his age.

The emaciation was so great that no person could recognize the countenance of the living in the dead body. On examination an immense excavation was found in the left lung, with tubercles and adhesions on the same side. The right lung also was studded with tubercles, some of them very hard, others beginning to soften down.

Few physicians, during their life time, have enjoyed the satisfaction of seeing their works so widely circulated, and their reputation so durably established. In his first and greatest work, the essay on Typhus, he has divided fever into three varieties, the simple, congestive and inflammatory—in the first of which, general reaction follows the stage of depression—the second, in which the circulation is so much impaired, during the cold stage, or the vital functions are so much oppressed by visceral congestion, that reaction cannot take place—the last, in which the fever is accompanied by inflammation, either produced by external causes anterior to or simultaneous with the general excitement; or arising during the course of the disease, from high vascular excitement acting upon organs, exposed to casual irritation, or suffering from the derangements of the circulation which have taken place during the cold stage. In his subsequent works, he has attempted to extend these principles of pathology to a variety of febrile diseases. Believing them to be correct, we have no disposition to compare them with the views of the French Pathologist, nor to dispute with the latter the exclusive merit of prescribing remedies upon rational principles. Convinced, however, that post mortem examinations can only shew the condition of diseased organs at the moment of death, we deny the right to infer from morbid anatomy the origin of fever ; and with a firm belief that these views are sustained by the order in which the symptoms succeed each other, we confidently expect that time will confirm the interpretation, which pathologists have hitherto almost uniformly given to the phenomena of fever.

But it would be doing injustice to the memory of Dr. Armstrong, to rest any portion of his reputation upon his speculative opinions. Indeed he has himself rather avoided them, treating them cursorily when they came in his way, rather as questions of curiosity than of practical importance. His descriptions of disease, clear, comprehensive and concise, indicate a mind capable of the most extended views in science, with a talent for the most minute and faithful investigation, and will be read with interest, when the disputes which now divide the medical world shall be forgotten.

The practice which he recommended was energetic and decided, perhaps to an extreme, combined, however, with that accurate investigation of symptoms, and that strict attention to subsidary remedies, which must certainly have made him a safe and successful practitioner. The similarity of many of his theoretical and practical views with those of the late Dr. Rush, cannot have escaped the observation of any person familiar with the works of the latter ; although the views of Rush, necessarily indefinite from his imperfect knowledge of the pathology of congestion, are often still further obscured by his peculiar, and often unintelligible speculations.

It is now, we believe, generally admitted, that the typhus fever of this country seldom calls for the decided practice recommended by Armstrong. Dr. Rush found that the yellow fever changed its character so much in succeeding years, that he was compelled to modify very materially the practice which he found so successful in 1793 ; and it is generally believed that this illustrious man lost his life, by attempting to apply the same practice to the typhoid epidemic, which prevailed so extensively in the United States a few years ago.

In private life, Dr. Armstrong is represented as extremely interesting—an eloquent companion, a generous rival, a warm and sincere friend. Very few would be inclined to suspect from the tenor of his writings, that he was much inclined to speculation or hypothesis ; yet in conversation and in his lectures, he is said to have embarked in discussions of this nature, with great zeal, and to have brought to the support of his views, a body of facts and illustrations which he used with much facility and address. The activity and ardor of his mind, however, disposed him to the observation and hasty generalization of facts, rather than to the profound investigation requisite for constructing a durable system. From this trait of character he was liable to fluctuate in his views; and under these circumstances, his candor led him to renounce his opinions as hastily as he had before adopted them. In his original essay on typhus, he made the fact of its arising from contagion a criterion of the disease. Subsequently he renounced this opinion, and considered typhus as a fever of malaria, disposed to assume the continued form, in consequence of the tendency to local inflammation. It is not so generally known that in after life, his practice became cautious and even timid, and that cold drawn castor oil was made to supply the place of calomel and the lancet. Perhaps this change may, in some degree, be attributed to the different rank which his patients occupied, in the later years of his life, they being probably drawn from the two extremes of society, and consequently requiring a more cautious treatment; but whatever may have been the cause, a prejudice thereby, was raised against him, which rendered him averse to similar discussions. The fact may afford a striking lesson to the intolerant partisans of speculative opinions, and a still more useful caution to those who, from their success, are disposed to decry the practice of those who prescribe upon different principles.

In reviewing his career, we are disposed to regret that, by extending his labours so far beyond the limits of prudence, he should have deprived the world of his services, just at the period when they were becoming most valuable. It may be urged, by those who estimate a man's usefulness by the result of his individual labours, that by attempting less he might have effected more ; that by directing his exertions to a more definite object, he might more effectually have advanced the interests of humanity, and have lived long, an ornament to his profession and a benefit to society. But when we reflect how few are willing to follow his example, and how many are unwilling to exert that labour and study which are necessary to qualify them for the emergencies of ordinary practice, our regrets may be diminished. The different branches of medicine are so intimately connected, that the accomplished physician must be familiar with the principles of them all. The temple of Medical fame has, by the united labours of numerous individuals, been raised to such an elevation, that he who would climb to the summit, must be urged to unceasing exertion by every motive that can actuate the human mind. As an individual extends his reputation, his influence and utility increase in a geometrical ratio ; and although the personal labours of Dr. Armstrong, have been terminated by death, he will live long to the profession, and especially to those who were within the reach of his personal influence, in his recorded labors, and the recollection of his bright example. J. C. F.

